# Regional and sex differences in retinal detachment surgery: Japan-retinal detachment registry report

**DOI:** 10.1038/s41598-021-00186-w

**Published:** 2021-10-18

**Authors:** Ryoh Funatsu, Hiroto Terasaki, Taiji Sakamoto, the Japan Retinal Detachment Registry study group, Shuichi Yamamoto, Takayuki Baba, Eiju Sato, Masayasu Kitahashi, Tomoaki Tatsumi, Gen Miura, Tomohiro Niizawa, Taiji Sakamoto, Keita Yamakiri, Toshifumi Yamashita, Hiroki Otsuka, Seiji Sameshima, Narimasa Yoshinaga, Shozo Sonoda, Akito Hirakata, Takashi Koto, Makoto Inoue, Kazunari Hirota, Yuji Itoh, Tadashi Orihara, Yoshinobu Emoto, Masahiko Sano, Hiroyuki Takahashi, Ryo Tokizawa, Hidetoshi Yamashita, Koichi Nishitsuka, Yutaka Kaneko, Katsuhiro Nishi, Akitoshi Yoshida, Shinji Ono, Hiroyuki Hirokawa, Kenji Sogawa, Tsuneaki Omae, Akihiro Ishibazawa, Shoji Kishi, Hideo Akiyama, Hidetaka Matsumoto, Ryo Mukai, Masahiro Morimoto, Mitsuru Nakazawa, Yukihiko Suzuki, Takashi Kudo, Kobu Adachi, Susumu Ishida, Kousuke Noda, Satoru Kase, Shohei Mori, Ryo Ando, Michiyuki Saito, Tomohiro Suzuki, Kanji Takahashi, Yoshimi Nagai, Tadashi Nakauchi, Haruhiko Yamada, Shunji Kusaka, Daishi Tsujioka, Akitaka Tsujikawa, Kiyoshi Suzuma, Tatsuro Ishibashi, Koh-Hei Sonoda, Yasuhiro Ikeda, Riichiro Kohno, Keijiro Ishikawa, Mineo Kondo, Maki Kozawa, Takashi Kitaoka, Eiko Tsuiki, Yuichiro Ogura, Munenori Yoshida, Hiroshi Morita, Aki Kato, Yoshio Hirano, Kazuhiko Sugitani, Hiroko Terasaki, Takeshi Iwase, Yasuki Ito, Shinji Ueno, Hiroki Kaneko, Norie Nonobe, Taro Kominami, Noriyuki Azuma, Tadashi Yokoi, Hiroyuki Shimada, Hiroyuki Nakashizuka, Takayuki Hattori, Ari Shinojima, Yorihisa Kitagawa, Fumio Shiraga, Yuki Morizane, Shuhei Kimura, Tsunehiko Ikeda, Teruyo Kida, Takaki Sato, Masanori Fukumoto, Kazuyuki Emi, Hiroshi Nakashima, Masahito Ohji, Masashi Kakinoki, Osamu Sawada, Shinobu Takeuchi, Sumiyoshi Tanaka, Tomohiro Iida, Hideki Koizumi, Ichiro Maruko, Taiji Hasegawa, Akiko Kogure, Hiroyuki Iijima, Tomohiro Oshiro, Yasushi Tateno, Wataru Kikushima, Atsushi Sugiyama, Seigo Yoneyama, Kazuaki Kadonosono, Shimpei Sato, Shin Yamane

**Affiliations:** 1 Department of Ophthalmology, Kagoshima University Graduate School of Medical and Dental Sciences, Kagoshima, Japan; 2 Chiba University, Chiba, Japan; 3 Kyorin University, Mitaka, Japan; 4 Yamagata University, Yamagata, Japan; 5 Asahikawa Medical University Hospital, Asahikawa, Japan; 6 Gunma University, Maebashi, Japan; 7 Hirosaki University, Hirosaki, Japan; 8 Hokkaido University, Sapporo, Japan; 9 Kansai Medical University Hospital, Hirakata, Japan; 10 Kindai University Sakai Hospital, Sakai, Japan; 11 Kyoto University, Kyoto, Japan; 12 Kyushu University, Fukuoka, Japan; 13 Mie University, Tsu, Japan; 14 Nagasaki University, Nagasaki, Japan; 15 Nagoya City University, Nagoya, Japan; 16 Nagoya University, Nagoya, Japan; 17 National Center for Child Health and Development, Tokyo, Japan; 18 Nihon University Hospital, Tokyo, Japan; 19 Okayama University, Okayama, Japan; 20 Osaka Medical School, Takatsuki, Japan; 21 Osaka Rosai Hospital, Sakai, Japan; 22 Shiga Medical University, Otsu, Japan; 23 Takeuchi Eye Clinic, Tokyo, Japan; 24 Tokyo Women’s Medical College, Tokyo, Japan; 25 Yamanashi University, Kofu, Japan; 26 Yokohama City University Medical Center, Yokohama, Japan

**Keywords:** Diseases, Health care, Medical research

## Abstract

It is known that social factors affect the choice of treatments, and special attention has been paid to sex differences. The purpose of this study was to determine whether regional and sex differences exist in the treatment of rhegmatogenous retinal detachment (RD). We used Japan-RD Registry database of 2523 patients aged ≥ 40 years between February 2016 and March 2017 in 5 Japanese regions. Regional differences of patients’ perioperative factors were analyzed. The factors affecting the proportion of patients who underwent surgery within one week of the onset, defined as early-surgery, were examined by logistic regression. We observed regional differences in perioperative factors, especially in the use of phacovitrectomy, general anesthesia, and air-tamponade, which was higher in certain regions. (Fisher’s exact test, all *P* = 0.012) The proportion of early-surgery was significantly higher among men in Kyushu region (Odds ratio (OR) 1.83; 95% confidence interval (CI) 1.08–3.12; *P* = 0.02), and it was also significantly higher after adjusting for covariates (OR 1.89; 95% CI 1.06–3.42; *P* = 0.02). Regional and sex differences exist in the treatment of RD in Japan. Although there was no significant differences in the anatomical outcomes, women in certain regions of Japan are less likely to receive early surgical intervention for RD.

## Introduction

The reasons for choosing a specific type of surgery in ophthalmology have changed with the advancement of surgical methods and drug therapies^1–3^. Although the eye and the patient's general condition have been prioritized in choosing the surgical methods, social factors frequently affect the decision in the real world. For example, in the USA, it has been reported that social factors are involved in addition to the preferences of patients and surgeons^4–9^. If the most effective medical care is altered by factors other than medical, it is necessary to improve them.

Non-medical factors influencing medical care include regional differences, education, socio-economic status, and ethnicity^10–18^. Regarding sex differences, there are reports of poorer postoperative outcomes in women with cardiovascular diseases and cerebral infarctions^11,13^. In ophthalmology, the poorer baseline findings at the initial treatment of female patients have been reported for diabetic macular edema and age-related macular degeneration^19,20^. It has been assumed that there were some biological factors in women and constitution to account for these differences.

It was recently reported in the USA that the ratio of patients who underwent surgery for rhegmatogenous retinal detachment (RD) was lower in women^4,5,21^. The waiting period for cataract surgery in Sweden was also longer for women than men, and a sex gap was suggested^22^. In Asia, there have been few analyses of eye care from the perspective of sex equality. Japan is a small country with a relatively uniform ethnicity. There is universal public health insurance, and the differences in the income and education levels among the residents is not large. Moreover, it is generally believed that standardized treatment was provided in medical teaching hospitals. However, it has not been determined whether there are significant differences in the treatment of patients with RD.

Thus, the purpose of this study was to determine whether social factors were affecting the treatment of RD. We paid particular attention to sex disparities and investigated this in Japan where sex inequality has been reported^23^.

## Results

### Regional differences

#### Preoperative factors

There were no significant differences in the age, sex, or laterality among the different regions of Japan (Table 1). Although there was no significant difference in the refractive error, there was a significant difference in the axial length (*P* = 0.013) and preoperative visual acuity (*P* < 0.001) in the different regions. There was also a significant difference in the intraocular pressure (IOP) among the different regions (*P* < 0.001), there was no significant difference in the percentage of patients with IOP less than 5 (*P* = 0.281). The percentage of patients undergoing early surgery was significantly different (*P* = 0.012), with the Kanto metropolitan region having the lowest percentage (Chubu 41%; Hokkaido-Tohoku 37%; Kanto 29%; Kinki 36%; Kyushu 39%). There were no significant differences between regions in the type of RD, location and size of the causative retinal break, presence of macular off detachment, presence of choroidal detachment, and presence of a giant tears (supplement Table S1 and S2). There were significant differences in the distribution of the type of retinal tears (*P* = 0.022), the extent of the RD (*P* = 0.025), and the distribution of PVR grade (*P* = 0.016), with more atrophic holes in the Kinki region, the largest extent of RD in the Kanto metropolitan-area, and the largest percentage of PVR grade C in the Kyushu region (Table 1 and supplement Table S2). The size of the retinal detachment was the largest in the Kanto metropolitan area and the percentage of PVR grade C was the highest in the Kyushu area.

**Table 1:** Preoperative background of subjects by regions.

Characteristics^3^	Regions											Adjusted p value^2^
	Chubu N = 198^1^		Hokkaido Tohoku N = 293^1^		Kanto N = 1111^1^			Kinki N = 620^1^		Kyushu N = 301^1^		
**Age (years)**	60.4 ± 10.2		61.8 ± 10.2		60.4 ± 10.4			60.7 ± 10.7		60.7 ± 10.9		> 0.999
**Sex (Female)**	66 (33%)		100 (34%)		349 (31%)			216 (35%)		110 (37%)		> 0.999
**Right**	120 (61%)		167 (57%)		606 (55%)			325 (52%)		170 (56%)		> 0.999
**Axial length (mm)**	25.58 ± 1.71		25.02 ± 1.84		25.59 ± 1.90			25.66 ± 2.01		25.47 ± 1.93		0.013
**Spherical equivalent (D)**	− 3.1 ± 4.2		− 3.0 ± 4.4		− 3.2 ± 4.5			− 2.6 ± 3.9		− 3.0 ± 4.4		0.281
**Intraocular pressure (mmHg)**	12.4 ± 3.7		12.7 ± 3.7		13.7 ± 3.6			12.9 ± 3.6		12.3 ± 3.6		< 0.001
**Intraocular pressure less than 5 mmHg**	8 (4.1%)		10 (3.4%)		16 (1.5%)			15 (2.4%)		10 (3.3%)		0.395
**BCVA (logMAR)**	0.57 ± 0.77		0.70 ± 0.89		0.53 ± 0.78			0.56 ± 0.80		0.74 ± 0.81		< 0.001
**Onset to Surgery (≤ 1 week)**	81 (41%)		107 (37%)		319 (29%)			221 (36%)		117 (39%)		0.012
**Lens status**												0.136
Aphakia	2 (1.1%)		0 (0%)		12 (1.3%)			3 (0.6%)		3 (1.2%)		
IOL (Intracapsular)	7 (4.0%)		5 (2.0%)		23 (2.5%)			11 (2.2%)		19 (7.4%)		
IOL (Intrascleral)	1 (0.6%)		2 (0.8%)		8 (0.9%)			2 (0.4%)		0 (0%)		
Phakia	164 (94%)		239 (97%)		888 (95%)			493 (97%)		234 (91%)		
**PVD**												0.012
+	170 (86%)		232 (79%)		951 (86%)			501 (81%)		259 (86%)		
Unknown	6 (3.0%)		33 (11%)		14 (1.3%)			24 (3.9%)		1 (0.3%)		
**RD (quadrant)**	2.03 ± 0.91		1.93 ± 0.89		2.07 ± 0.88			1.92 ± 0.86		1.96 ± 0.93		0.025
**Macular detachment**												> 0.999
Macula off	91 (46%)		145 (49%)		519 (47%)			277 (45%)		158 (52%)		
Unknown	0 (0%)		0 (0%)		10 (0.9%)			5 (0.8%)		2 (0.7%)		
**PVR (grade)**												0.016
N/A	183 (92%)		278 (95%)		1034 (93%)			541 (87%)		264 (88%)		
B	12 (6.1%)		8 (2.7%)		49 (4.4%)			51 (8.2%)		16 (5.3%)		
C	3 (1.5%)		7 (2.4%)		28 (2.5%)			28 (4.5%)		21 (7.0%)		
**Choroidal detachment**	9 (4.5%)		7 (2.4%)		38 (3.4%)			26 (4.2%)		23 (7.6%)		0.228
**Giant tear**	24 (12%)		28 (9.7%)		89 (8.1%)			42 (6.9%)		24 (8.0%)		> 0.999

^1^Mean ± SD; n (%).

^2^Kruskal-Wallis rank sum test for Continuous data; Fisher's Exact Test for Count Data with simulated P value. Holm correction for multiple testing.

^3^BCVA: best corrected visual acuity, IOL: intraocular lens, PVD: posterior vitreous detachment, RD: retinal detachment, PVR: Proliferative vitreoretinopathy.

#### Operative factors

Statistically significant differences were observed in the surgical techniques, gauge of instruments used, percentage of eyes that underwent phacovitrectomy, tamponade material used, and anesthesia technique among the different regions of Japan (each adjusted *P* = 0.012; Table 2). The Kyushu region had the highest rate of PPV, the Hokkaido and Tohoku regions had the highest rate of SB, and the Kanto-metropolitan region had the highest rate of PPV + SB. The rate of phacovitrectomy was highest in the Kyushu and Hokkaido/Tohoku regions. Air tamponade was the most commonly used in the Kanto and Kinki regions, and silicone oil was the most commonly used in the Kyushu region.

**Table 2:** Operative background of subjects by regions.

Characteristics	Regions					Adjusted p value^2^
	Chubu N = 198^1^	Hokkaido Tohoku N = 293^1^	Kanto N = 1111^1^	Kinki N = 620^1^	Kyushu N = 301^1^	
**Operative method**						0.012
PPV	165 (83%)	210 (72%)	863 (78%)	527 (85%)	261 (87%)	
PPV + SB	7 (3.5%)	15 (5.1%)	90 (8.1%)	16 (2.6%)	10 (3.3%)	
SB	26 (13%)	68 (23%)	158 (14%)	77 (12%)	30 (10.0%)	
**Gauge**						0.012
20	0 (0%)	1 (0.5%)	10 (1.1%)	0 (0%)	0 (0%)	
23	0 (0%)	1 (0.5%)	6 (0.6%)	26 (4.9%)	8 (2.9%)	
25	166 (99%)	214 (98%)	853 (90%)	486 (92%)	269 (97%)	
27	2 (1.2%)	2 (0.9%)	75 (7.9%)	14 (2.7%)	0 (0%)	
**Combined cataract surgery**^**3**^	104 (76%)	162 (96%)	563 (82%)	384 (94%)	189 (96%)	0.012
**Tamponade material**						0.012
Air	6 (3.4%)	27 (11%)	201 (21%)	116 (21%)	9 (3.3%)	
SF6	140 (80%)	207 (86%)	640 (66%)	394 (72%)	225 (83%)	
C3F8	9 (5.1%)	0 (0%)	24 (2.5%)	18 (3.3%)	0 (0%)	
Silicone oil	20 (11%)	7 (2.9%)	49 (5.0%)	23 (4.2%)	38 (14%)	
Others	0 (0%)	0 (0%)	63 (6.4%)	0 (0%)	0 (0%)	
**Anesthesia type**						0.012
General anesthesia	2 (1.0%)	28 (9.6%)	7 (0.8%)	8 (1.3%)	39 (13%)	
Local anesthesia	196 (99%)	265 (90%)	899 (99%)	612 (99%)	262 (87%)	

^1^n (%).

^2^Fisher's Exact Test for Count Data with simulated p-value. Holm correction for multiple testing.

^3^Among phakic patients who got vitrectomy without scleral buckle.

### Failure proportion in six months

There was no regional difference in the failure proportion at 6 months (*P* > 0.999; supplement Table S3).

### Factors associated with interval from onset to surgery

The variables selected by backward forward stepwise analyses were the regions, age, axial length, type of retinal break, location of retinal break, macula-off, choroidal detachment, and giant tear. Multiple logistic regression analysis showed that the factors such as region, age, axial length, type of tear, and choroidal detachment were significantly correlated with the rate of early surgery (maximum *P* = 0.023; Table 3). Compared to the Kanto region, the percentage of patients who underwent early surgery was higher in the Kyushu, Kinki, and Chubu regions. Older patients and eyes with choroidal detachment were less likely to receive early surgery. Eyes with longer axial length were more likely to receive early surgery.

**Table 3:** Factors associated with the days from onset to surgery in Japan by multiple logistic regression.

Characteristics	Coefficients	Odds ratio	*P* value
**Regions (reference: Kanto)**			
Hokkaido Tohoku	0.16	1.78	0.448
Chubu	0.63	1.87	0.002
Kinki	0.47	1.60	< 0.001
Kyushu	0.67	1.95	< 0.001
**Age**	− 0.02	0.98	0.011
**Axial length**	0.18	1.19	< 0.001
**Retinal break types (reference: Tear)**			
Retinal holes, atrophic hole, or retinal atrophy with lattice degeneration	− 1.04	0.35	< 0.001
Breaks at/near the vireous base	− 0.76	0.47	0.169
Macula hole	− 2.65	0.07	0.023
Unknown	− 1.35	0.26	0.213
**Retinal break location (reference: Inferior Nasal)**			
Inferior temporal	− 0.13	0.88	0.609
Superior temporal	0.40	1.50	0.077
Superior nasal	0.27	1.31	0.269
Posterior pole	0.48	1.62	0.604
**Macula detachment (reference: Macula off)**			
Macula on	0.17	1.19	0.158
Unknown	− 13.39	< 0.01	0.975
**Choroidal detachment**	− 1.26	0.28	0.022
**Giant tear**	0.29	1.34	0.136

### Sex differences in interval to surgery by regions

**Figure 1:**
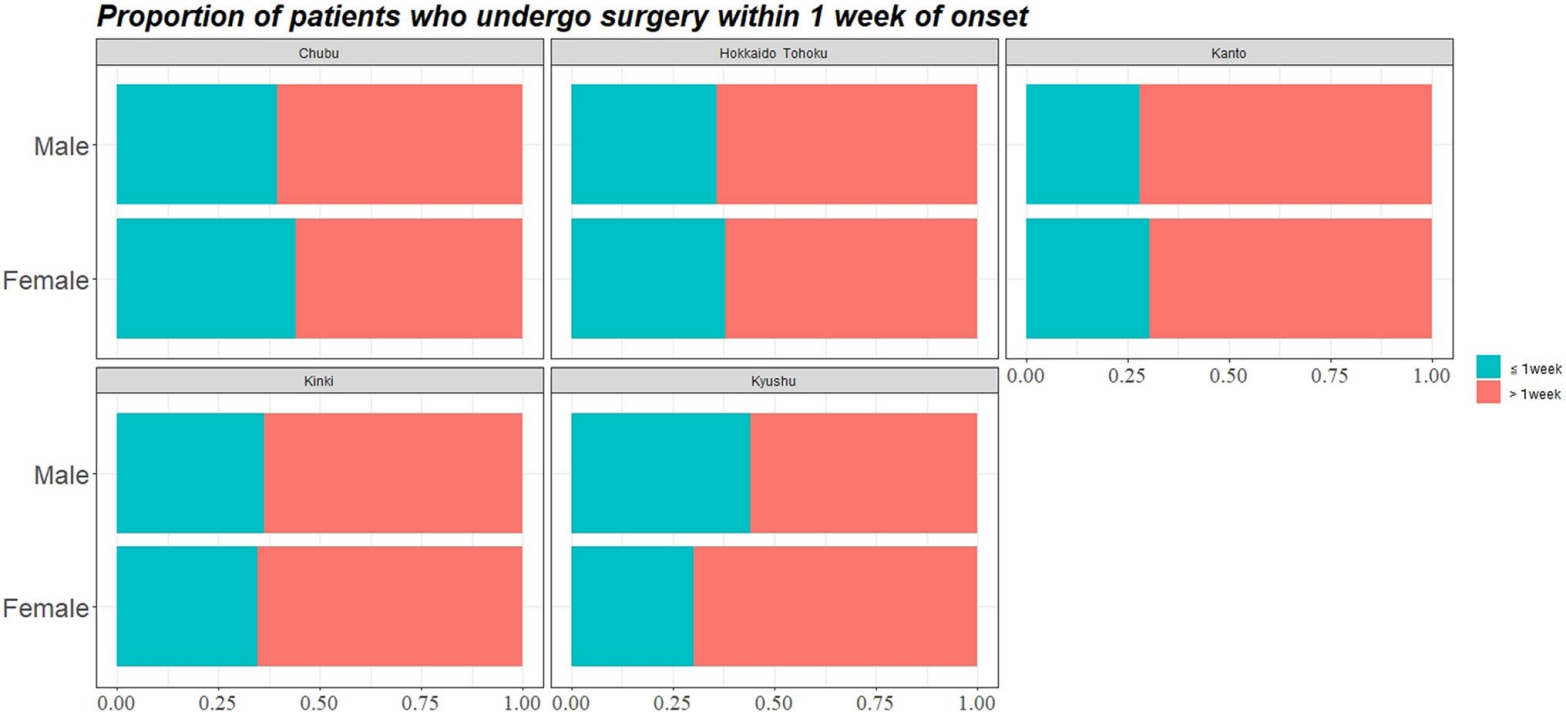
Comparison of the proportion of patients who undergo surgery within 1 week of onset between genders by regions.

There was no sex difference in the rate of early surgery in Japan as a whole. (Odds ratio = 0.99) When compared by regions, the rate was statistically and significantly higher in men than in women only in the Kyushu region (Odds ratio = 1.83; 95% confidence interval 1.08 – 3.12), and no significant differences were observed in the other regions (Fig. 1 and Table 4). In examination of the association between the variables selected by the stepwise method described above plus sex and early surgery in the Kyushu region, sex (*P* = 0.032) and axial length (*P* = 0.008) were each statistically and significantly correlated (Table 5).

**Table 4:** Odds ratio between genders of undergoing surgery within 1 week of onset and the results of statistical test.

Regions	Odds ratio^2^	*P* value^1^
All regions	0.99 (95% CI 0.83–1.19)	0.964
Chubu	0.83 (95% CI 0.44–1.58)	0.544
Hokkaido Tohoku	0.91 (95% CI 0.54–1.55)	0.703
Kanto	0.89 (95% CI 0.67–1.19)	0.432
Kinki	1.06 (95% CI 0.74–1.53)	0.792
Kyushu	1.83 (95% CI 1.08–3.12)	0.020

^1^Fisher's Exact Test for Count Data with simulated *P* value.

^2^CI: confidence interval.

**Table 5:** Factors focusing on sex differences associated with the days from onset to surgery in Kyushu region analyzed by multiple logistic regression.

Characteristics	Coefficients	Odds ratio	*P* value
**Sex (reference: male)**	− 0.64	1.89	0.032
**Age**	0.00	1.00	0.959
**Axial length**	0.23	1.25	0.008
**Retinal break types (reference: Tear)**			
Retinal holes, atrophic hole, or retinal atrophy with lattice degeneration	− 0.84	0.43	0.078
Breaks at/near the vitreous base	0.38	1.46	0.634
Macula hole	− 0.51	0.60	0.757
Unknown	− 1.47	< 0.01	0.987
**Retinal break location (reference: Inferior Nasal)**			
Inferior temporal	− 0.51	0.60	0.413
Superior temporal	0.19	1.21	0.732
Superior nasal	− 0.15	0.86	0.802
Posterior pole	− 0.76	0.47	0.615
**Macula detachment (reference: Macula off)**			
Macula on	0.45	1.57	0.112
Unknown	− 14.08	< 0.01	0.987
**Choroidal detachment**	− 1.21	0.30	0.091
**Giant tear**	− 0.05	0.95	0.921

## Discussion

It has been reported in the USA that there are differences in the quality of medical care by region which is understandable considering the differences in the health insurance systems, regional socio-industrial structures, and racial composition^24–26^. In fact, the contribution of the provider level and regional level to the selection of treatment for RD has been shown for the United States^5^. Japan is a relatively small island nation with a homogeneous population, standardized medical education, and universal public health insurance. However, the results of this study suggested that regional differences exist in treatment strategies including the choice of anesthesia, surgical instruments, and surgical techniques in Japan as well.

Among the preoperative factor, the axial length was the longest in the Kinki region, but the difference in mean values between the shortest region with the shortest axial length was in the Hokkaido/Tohoku region of about 0.6 mm. Thus, there was no clinically meaningful difference. There was a regional difference only in the type of retinal breaks with a higher percentage of atrophic hole in the Kinki region. In a previous report using this registry data, an atrophic hole was a significant factor in selecting SB surgery^27^. If so, the Kinki region should have had a higher percentage of SB, but on the other hand, it had a higher percentage of PPV. There are complex factors involved in the surgical selection in the actual clinical practice, and preoperative ocular findings alone may not produce regional differences in the selection of PPV and SB.

The patients in the Kanto region had the largest quadrants of detached retina and the lowest rate of early surgery. This relationship is reasonable because the area of detached retina become larger with the interval from the onset of RD to the time of surgery. The Kyushu region is one of the farthest from the Kanto-metropolitan area and tends to be mainly relatively rural areas than the other regions, so the residents in this region might have less access to clinics. However, the time to surgery was shorter in the Kyushu region than in the Kanto. In the Kanto region, there are many small clinics and there are fewer direct referrals to surgical facilities^28^. This may explain why it took longer to get to surgery. In other words, it is possible that there is a problem with the medical system rather than a problem with transportation access.

We paid special attention to the time from the onset of the symptoms to the time of surgery. Because there were variations in the preoperative factors among the regions, we examined the factors that affected the rate of surgery within 1 week of onset and adjusted for them. However, even after the adjustments, there were regional differences in the time to surgery. These findings suggested that socioeconomic factors such as the number of specialized retinal clinics, transportation access, and economic factors may be involved in the time to surgery, making it difficult to compare and interpret regional differences.

On the other hand, an important result was obtained regarding the differences between the sexes in the same region. As reported in the USA, when the time from onset of disease to surgery (within a week or later) was used to determine whether women tended to receive later treatment, there was no difference in Japan as a whole. Nonetheless, the rate was significantly higher for men only in the Kyushu region even after adjusting for the preoperative factor. It has been suggested that sex disparity may be present in emergency medical services, and this has been an issue worldwide. Thus, Maintz et al. reported that in acute ischemic stroke, women had a longer time from onset to arrival at the stroke clinic^14^. Park et al. reported a sex difference in the treatment of RD in Korea^29^. However, they stated that the reason of PPV being more common in men is due to the fact that traumatic RD is more common in men, and they did not consider social factors such as sex disparities^29^.

Patients with traumatic RD would present earlier. Although there were only 4 (2.1%) cases with traumatic RD for men and 1 (0.9%) for women and they tended to be more common for men in the Kyushu region, the difference was not that large (supplement Table S4). Moreover, in our study, having a macular hole RD lowered the probability of surgery within one week of the onset in our study (Odds ratio = 0.07, Table 3), and women tended to have more macular hole RD and less retinal tear than men in Kyushu region (supplement Table S4 and S5). These biologic differences may also influence the time for having delayed surgery in women. However, even in the analysis that adjusted for all factors related to time to surgery, sex was still a factor that affected the delay to surgery, suggesting that unknown or social factors may be involved (Table 5).

Kyushu is an economically poor region of Japan and has a traditionally conservative culture with a male-dominated society with a low sex equity index^30^. It would be important that there is a sex gap in health care access only in this region. This does not immediately prove that this is a result of sexual inequality, but this possibility of sex disparity cannot be denied^30^. Although a patient with traumatic RD may come earlier to the clinic and the one with traumatic RD tended to be more common in men in the Kyushu region, it should be noted that the effect of sex was significant even after we adjusted this effect statistically (supplement Table S4, supplement Table S5 and Table 5).

Importantly, there were no regional or sex differences in the postoperative anatomical outcomes in any region of Japan. On the other hand, it is possible that this is why the sex gap was overlooked. Because our study did not include data on important factors such as the visual acuity and long-term outcomes, we cannot say that there was no real sex gap in the treatment outcomes.

There are some strengths in this study. The number of RD surgeries included in our data is the most detailed and comprehensive data on RD performed in Japan in 2016. All the results were obtained from teaching hospitals that met the criteria, a group of hospitals that were guaranteed to be the most guideline-compliant in terms of choosing surgical methods including the timing of surgery. The data acquisition and registration were done by retina specialists who met the JRVS criteria.

There are limitations in this study. First, this was a retrospective study and does not represent all the data in a country. Second, we do not have information on the social background of the patients, such as the distance from their residence to the hospital, annual income, occupation, education, race, pre-existing conditions, and activities of daily living. In addition, because the time from onset of symptoms to surgery was based on the patient's memory, the existence of recall bias cannot be denied. The interval between the initial diagnosis of RD and the surgery was also not definitively known. Finally, we analyzed the relationship between the anatomical outcome and social factors in the RD patients, not the ones between the functional outcomes such as the perioperative visual acuity. We chose the former as reported^31,32^. In addition, we chose it because the preoperative visual acuity strongly affects the postoperative visual acuity^33^, and our preliminary analysis showed preoperative visual acuity varied significantly among the regions (Table 1).

In conclusion, the results of our study showed that there are regional differences in the treatment of RD in Japan. Female patients with RD have a longer time to surgery than men in the Kyushu region. The time to surgery for RD is influenced by a variety of factors but sex disparity has not been well studied in Asia due to lack of interest. If this exists, it is a medical problem that needs to be solved soon.

## Methods

### Study design and registered data

The details of the study design have been published in detail elsewhere^34^. This is a private database of Japan retina and vitreous society (JRVS), and the requirement for individual written informed consent from the patients was approved to be waived by all the hospitals or institutes except for the Kyushu University hospital ethical committee. For the participants of Kyushu University, written informed consent was obtained from all the participants. The datasets used during the current study are available from the JRVS on request. Surgical data on the consecutive cases of RRD performed by vitreoretinal specialists certified by the Japanese Ophthalmological Association were collected from 26 ophthalmological institutions in the Hokkaido/Tohoku, Kanto, Chubu, Kinki, Chugoku, and Kyushu regions through this database project. Each region is considered to historically be very similar cultural-industrial zones, and they still share common socio-cultural characteristics. The data were collected online through a website between February 2016 and May 2017. Data were collected on more than 50 preoperative, operative, and postoperative factors for up to 6 months postoperatively as reported^3,33–35^. The information collected was not personally identifiable, and details of obtaining research consent have been published^34^.

### Subjects and exclusion criteria

**Figure 2:**
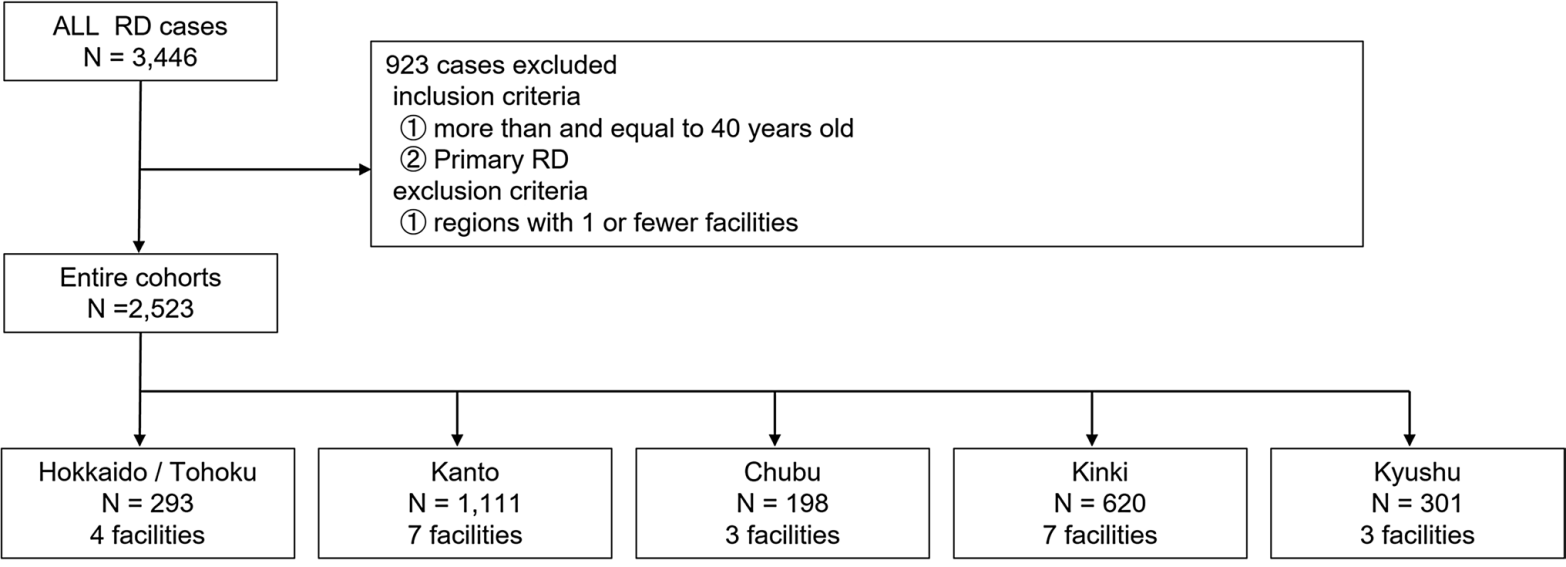
Flow chart describing selecting the study population. RD, retinal detachment.

The details of the patients’ demographics have been published^34^. The number of cases in the database was 3446 from which 2595 cases of first retinal detachment surgery who were ≥ 40 years of age (Fig. 2). The regions were determined based on the classification of statistical data published by the Japan Cabinet Office, and the Chugoku and Shikoku regions with one or less participating institution were excluded, resulting in 2523 cases. Based on the above criteria, the number of cases and facilities used in the analyses for each region was 293 cases and 4 facilities in the Hokkaido and Tohoku regions, 1111 cases and 7 facilities in the Kanto region, 198 cases and 3 facilities in the Chubu region, 620 cases and 7 facilities in the Kinki region, and 301 cases and 3 facilities in the Kyushu region.

### Statistical analyses

The definition of success at 6 months was based on the European Vitreo-Retinal Society (EVRS) study as reported^36^. The perioperative factors were compared among the following regions: Hokkaido/Tohoku, Kanto, Chubu, Kinki, and Kyushu. The means and standard deviations are reported for continuous variables and numbers and percentage are reported for categorical variables. The presence or absence of combined cataract surgery was examined in a population limited to pars plana vitrectomy without scleral buckling in phakic eyes. Kruskal–Wallis rank sum test was used for continuous variables and Fisher's exact test for categorical variables and reported *P* values adjusted for multiple comparisons using the Holm correction.

### Definition of early surgery

The onset of the RD was based the time of onset of the symptoms as reported by the patient. We defined early surgery as surgery performed within 1 week of the self-reported onset date, and late surgery as surgery performed after that date. Multiple logistic regression analysis was used to examine the factors that influenced whether surgery was early or late, and the odds ratio and *P* values of each item are reported. For each region of Japan, the odds ratio and *P*-value of the differences in the proportion of early surgery between men and women were determined using Fisher's exact test. For the regions that the gender difference was significant, we used multiple logistic regression to examine the factors that affect the proportion of early surgery to calculate the odds ratio and *P* values for each item. We chose the independent variables for the regression analysis using the variables determined to significantly affect the proportion of early surgery by the multiple logistic regression analysis for all regions, plus sex variable. The cutoff for statistical significance was set at *P* < 0.05. R software (version 4.0.5) was used for all analyses. (https://www.r-project.org/).

### Ethical approval

This study was approved by the Ethics Committee of Kagoshima University (140093, 28-38) and was conducted in accordance with the Declaration of Helsinki^34^. All collected data were used in accordance with the Ethical Guidelines for Medical and Health Research Involving Human Subjects in Japan involving Human Subjects in Japan (https://www.mhlw.go.jp/file/06-Seisakujouhou-10600000-Daijinkanboukouseikagakuka/0000080278.pdf).

### Patient consent for publication

This study was an observational study based on the information collected as a result of standard care and identifiable information was de-identified in this registry study, the requirement for individual written informed consent from the patients was approved to be waived by all the hospitals or institutes except for the Kyushu University hospital ethical committee. For the participants of Kyushu University, written informed consent was obtained from all the participants.

## Supplementary Information

Supplementary Table S1.

Supplementary Table S2.

Supplementary Table S3.

Supplementary Table S4.

Supplementary Table S5.

## Data Availability

The datasets used during the current study are available from the corresponding author on request. The detailed protocol are published in our earlier paper. Sakamoto T, et al. Japan-Retinal Detachment Registry Report I: preoperative findings in eyes with primary retinal detachment. Jpn J Ophthalmol 2020. https://doi.org/10.1007/s10384-019-00702-6.
