# Supplementary material for: Regional and sex differences in retinal detachment surgery: Japan-retinal detachment registry report
Source: Sci Rep. 2021 Oct 18;11:20611. doi: 10.1038/s41598-021-00186-w (PMC8523544; doi:10.1038/s41598-021-00186-w)
Supplement: Supplementary file 1 — Supplementary Table S1. [file 41598_2021_186_MOESM1_ESM.docx]

supplement table 1. Retinal detachment types of subjects by regions.

|  | Regions | | | | |  |
| --- | --- | --- | --- | --- | --- | --- |
| Characteristics^3^ | Chubu  N = 198^1^ | Hokkaido Tohoku  N = 293^1^ | Kanto  N = 1,111^1^ | Kinki  N = 620^1^ | Kyushu  N = 301^1^ | Adjusted　p value^2^ |
| **Retinal detachment (types)** |  |  |  |  |  | 0.395 |
| Retinal tears related to traction | 154 (78%) | 235 (80%) | 907 (82%) | 471 (76%) | 223 (74%) |  |
| Retinal holes, atrophic hole, or retinal atrophy with lattice degeneration | 18 (9.1%) | 26 (8.9%) | 81 (7.3%) | 86 (14%) | 34 (11%) |  |
| Retinal breaks related to atopic dermatitis | 1 (0.5%) | 0 (0%) | 7 (0.6%) | 2 (0.3%) | 4 (1.3%) |  |
| Retinal breaks at the vitreous base | 1 (0.5%) | 0 (0%) | 3 (0.3%) | 0 (0%) | 0 (0%) |  |
| A macular hole in highly myopic eyes | 6 (3.0%) | 9 (3.1%) | 31 (2.8%) | 19 (3.1%) | 14 (4.7%) |  |
| A macular hole without highly myopic eyes | 1 (0.5%) | 2 (0.7%) | 1 (<0.1%) | 3 (0.5%) | 1 (0.3%) |  |
| Hereditary retinal detachment | 0 (0%) | 0 (0%) | 1 (<0.1%) | 1 (0.2%) | 0 (0%) |  |
| Post-cataract surgery | 7 (3.5%) | 11 (3.8%) | 33 (3.0%) | 12 (1.9%) | 7 (2.3%) |  |
| Trauma | 4 (2.0%) | 2 (0.7%) | 19 (1.7%) | 11 (1.8%) | 5 (1.7%) |  |
| Others | 6 (3.0%) | 8 (2.7%) | 28 (2.5%) | 15 (2.4%) | 13 (4.3%) |  |
| ^1^ n (%)  ^2^ Fisher's Exact Test for Count Data with simulated P value. Holm correction for multiple testing | | | | | | |
