# Supplementary material for: Regional and sex differences in retinal detachment surgery: Japan-retinal detachment registry report
Source: Sci Rep. 2021 Oct 18;11:20611. doi: 10.1038/s41598-021-00186-w (PMC8523544; doi:10.1038/s41598-021-00186-w)
Supplement: Supplementary file 2 — Supplementary Table S2. [file 41598_2021_186_MOESM2_ESM.docx]

supplement table 2. Characteristics of retinal breaks of subjects by regions.

|  | Regions | | | | |  |
| --- | --- | --- | --- | --- | --- | --- |
| Characteristics^3^ | Chubu  N = 198^1^ | Hokkaido Tohoku  N = 293^1^ | Kanto  N = 1,111^1^ | Kinki  N = 620^1^ | Kyushu  N = 301^1^ | Adjusted p value^2^ |
| **Retinal break (types)** |  |  |  |  |  | 0.022 |
| Tears | 162 (82%) | 250 (85%) | 955 (86%) | 485 (78%) | 237 (79%) |  |
| Atrophic holes | 26 (13%) | 30 (10%) | 90 (8.1%) | 101 (16%) | 37 (12%) |  |
| Breaks at/near the vitreous base | 4 (2.0%) | 3 (1.0%) | 27 (2.4%) | 11 (1.8%) | 11 (3.7%) |  |
| Macula hole | 6 (3.0%) | 8 (2.7%) | 31 (2.8%) | 21 (3.4%) | 14 (4.7%) |  |
| Unknown | 0 (0%) | 2 (0.7%) | 8 (0.7%) | 2 (0.3%) | 2 (0.7%) |  |
| **Retinal break (location)** |  |  |  |  |  | >0.999 |
| Inferior-Nasal | 16 (8.1%) | 16 (5.5%) | 69 (6.2%) | 53 (8.5%) | 20 (6.6%) |  |
| Inferior-Temporal | 24 (12%) | 38 (13%) | 180 (16%) | 108 (17%) | 52 (17%) |  |
| Posterior pole | 9 (4.5%) | 9 (3.1%) | 37 (3.3%) | 26 (4.2%) | 17 (5.6%) |  |
| Superior-Nasal | 42 (21%) | 59 (20%) | 252 (23%) | 140 (23%) | 67 (22%) |  |
| Superior-Temporal | 107 (54%) | 171 (58%) | 572 (52%) | 293 (47%) | 145 (48%) |  |
| **Tear size (degree)** |  |  |  |  |  | >0.999 |
| 0-30 | 172 (87%) | 260 (89%) | 1,009 (91%) | 568 (92%) | 276 (92%) |  |
| 30-60 | 22 (11%) | 27 (9.2%) | 76 (6.8%) | 38 (6.1%) | 21 (7.0%) |  |
| 60-90 | 2 (1.0%) | 5 (1.7%) | 13 (1.2%) | 11 (1.8%) | 1 (0.3%) |  |
| 90- | 2 (1.0%) | 1 (0.3%) | 12 (1.1%) | 3 (0.5%) | 3 (1.0%) |  |
| ^1^n (%)  ^2^Fisher's Exact Test for Count Data with simulated P value. Holm correction for multiple testing | | | | | | |
