# Supplementary material for: Regional and sex differences in retinal detachment surgery: Japan-retinal detachment registry report
Source: Sci Rep. 2021 Oct 18;11:20611. doi: 10.1038/s41598-021-00186-w (PMC8523544; doi:10.1038/s41598-021-00186-w)
Supplement: Supplementary file 3 — Supplementary Table S3. [file 41598_2021_186_MOESM3_ESM.docx]

supplement table 3. Failure proportion in six months by regions

|  | Regions | | | | |  |
| --- | --- | --- | --- | --- | --- | --- |
| Characteristics | Chubu,  N = 198 | Hokkaido Tohoku,  N = 293 | Kanto,  N = 1,111 | Kinki,  N = 620 | Kyushu,  N = 301 | Adjusted　ｐ value2 |
| **Failure proportions in six months** | 21 (11%) | 25 (11%) | 71 (7.5%) | 44 (7.7%) | 16 (6.3%) | **>0.999** |
| ^1^n (%)  ^2^Fisher's Exact Test for Count Data with simulated P value. Holm correction for multiple testing. | | | | | | |
