# Supplementary material for: Regional and sex differences in retinal detachment surgery: Japan-retinal detachment registry report
Source: Sci Rep. 2021 Oct 18;11:20611. doi: 10.1038/s41598-021-00186-w (PMC8523544; doi:10.1038/s41598-021-00186-w)
Supplement: Supplementary file 4 — Supplementary Table S4. [file 41598_2021_186_MOESM4_ESM.docx]

supplement table 4. Pre-operative background of subjects by gender in Kyushu

|  | Gender | |  |
| --- | --- | --- | --- |
| Characteristics | Male, N = 191^1^ | Female, N = 110^1^ | Adjusted p value^2^ |
| **Age (yr)** | 60.17 ± 11.15 | 61.65 ± 10.33 | >0.999 |
| **Axial length (mm)** | 25.57 ± 1.78 | 25.30 ± 2.17 | 0.939 |
| **Spherical equivalent (D)** | -2.88 ± 4.31 | -3.30 ± 4.67 | >0.999 |
| **Intraocular pressure (mmHg)** | 12.15 ± 3.60 | 12.53 ± 3.58 | >0.999 |
| **Intraocular pressure less than 5mmHg** | 6 (3.1%) | 4 (3.6%) | >0.999 |
| **BCVA (logMAR)** | 0.71 ± 0.79 | 0.77 ± 0.86 | >0.999 |
| **Lens status** |  |  | >0.999 |
| Aphakia | 2 (1.3%) | 1 (1.0%) |  |
| IOL (Intracapsular) | 13 (8.3%) | 6 (6.1%) |  |
| Phakia | 142 (90%) | 92 (93%) |  |
| **PVD** |  |  | >0.999 |
| + | 167 (87%) | 92 (84%) |  |
| Unknown | 0 (0%) | 1 (0.9%) |  |
| **Retinal detachment (types)** |  |  | 0.009 |
| A macular hole in highly myopic eyes | 0 (0%) | 14 (13%) |  |
| A macular hole without highly myopic eyes | 0 (0%) | 1 (0.9%) |  |
| Post-cataract surgery | 7 (3.7%) | 0 (0%) |  |
| Retinal breaks related to atopic dermatitis | 3 (1.6%) | 1 (0.9%) |  |
| Retinal holes, atrophic hole, or retinal atrophy with lattice degeneration | 24 (13%) | 10 (9.1%) |  |
| Retinal tears related to traction | 145 (76%) | 78 (71%) |  |
| Trauma | 4 (2.1%) | 1 (0.9%) |  |
| Others | 8 (4.2%) | 5 (4.5%) |  |
| **RD (quadrant)** | 2.02 ± 0.88 | 1.85 ± 1.00 | 0.456 |
| **Macular detachment** |  |  | >0.999 |
| Macula off | 102 (53%) | 56 (51%) |  |
| Unknown | 2 (1.0%) | 0 (0%) |  |
| **PVR (grade)** |  |  | >0.999 |
| B | 11 (5.8%) | 5 (4.5%) |  |
| C | 17 (8.9%) | 4 (3.6%) |  |
| **Choroidal detachment** | 14 (7.3%) | 9 (8.2%) | >0.999 |
| ^1^Mean ± SD; n (%)  ^2^Kruskal-Wallis rank sum test for Continuous data; Fisher's Exact Test for Count Data with simulated P value. Holm correction for multiple testing  ^3^BCVA: best corrected visual acuity, IOL: intraocular lens, PVD: posterior vitreous detachment, RD: retinal detachment, PVR:  Proliferative vitreoretinopathy | | | |
