# Supplementary material for: Regional and sex differences in retinal detachment surgery: Japan-retinal detachment registry report
Source: Sci Rep. 2021 Oct 18;11:20611. doi: 10.1038/s41598-021-00186-w (PMC8523544; doi:10.1038/s41598-021-00186-w)
Supplement: Supplementary file 5 — Supplementary Table S5. [file 41598_2021_186_MOESM5_ESM.docx]

supplement table 5. The characteristics of retinal break by gender in Kyushu

|  | Gender | |  |
| --- | --- | --- | --- |
| Characteristics | Male, N = 191^1^ | Female, N = 110^1^ | Adjusted p value^2^ |
| **Retinal break (types)** |  |  | 0.009 |
| Atrophic holes | 23 (12%) | 14 (13%) |  |
| Breaks at/near the vitreous base | 8 (4.2%) | 3 (2.7%) |  |
| Macula hole | 0 (0%) | 14 (13%) |  |
| Tears | 158 (83%) | 79 (72%) |  |
| Unknown | 2 (1.0%) | 0 (0%) |  |
| **Retinal break (location)** |  |  | 0.009 |
| Inferior-Nasal | 16 (8.4%) | 4 (3.6%) |  |
| Inferior-Temporal | 38 (20%) | 14 (13%) |  |
| Posterior pole | 2 (1.0%) | 15 (14%) |  |
| Superior-Nasal | 43 (23%) | 24 (22%) |  |
| Superior-Temporal | 92 (48%) | 53 (48%) |  |
| **Tear size (degree)** |  |  | >0.999 |
| 0-30 | 170 (89%) | 106 (96%) |  |
| 30-60 | 17 (8.9%) | 4 (3.6%) |  |
| 60-90 | 1 (0.5%) | 0 (0%) |  |
| 90- | 3 (1.6%) | 0 (0%) |  |
| **Giant tear** | 20 (11%) | 4 (3.6%) | 0.632 |
| ^1^n (%)  ^2^Fisher's Exact Test for Count Data with simulated P value. Holm correction for multiple testing | | | |
